# Tumoral Calcinosis of the Neck in a Patient with Systemic Sclerosis

**DOI:** 10.7759/cureus.3585

**Published:** 2018-11-13

**Authors:** Constantine N Logothetis, N. Suzanne Emil, Antonios H Tzamaloukas, Konstantin N Konstantinov

**Affiliations:** 1 Internal Medicine, University of New Mexico School of Medicine, Albuquerque, USA

**Keywords:** scleroderma, tumoral calcinosis

## Abstract

Tumoral calcinosis (TC) is rare in patients with systemic sclerosis but is associated with morbidity. Paraspinal TC may cause severe pain and potentially devastating neurological deficits. Surgical decompression by removing the TC masses and applying surgical techniques to support the spine have provided substantial relief of the symptoms in the majority of cases. However, death has occurred in the immediate postoperative period and can even occur after several months. Current indications for surgery include intractable neck pain and, most importantly, the development of neurological deficits. We present a patient with systemic sclerosis and symptomatic paraspinal TC in the neck treated conservatively for two years. This case report illustrates conditions permitting the sustained conservative treatment of paraspinal TC in systemic sclerosis patients.

## Introduction

The key pathophysiologic changes characterizing systemic sclerosis are the formation of specific autoantibodies, small vessel vasculopathy, and dysfunction of fibroblasts, causing an increased deposition of extracellular matrix [1]. The current criteria for classifying the disease are based on its main clinical and laboratory manifestations and include skin thickening, puffy fingers, finger-tip ulcers or pitting scars, telangiectasia, abnormal nail-fold capillary pattern, pulmonary arterial hypertension, interstitial lung disease, Raynaud’s phenomena, and scleroderma-specific antibodies. This last criterion includes anti-centromere antibodies, anti-topoisomerase (anti-Scl 70) antibodies, and anti-RNA polymerase III antibodies [1].

Calcinosis (soft tissue calcification) develops in connective tissue diseases, mainly systemic sclerosis, systemic lupus erythematosus, and dermatomyositis, as a result of the deposition of calcium salts in diseased tissues and causes significant clinical manifestations, including atrophy of the tissues involved, pain, and skin ulcers [2]. The subcutaneous tissues are the main sites of calcinosis in systemic sclerosis, but internal organs and muscles can also be involved [3]. Tumoral calcinosis (TC) is encountered infrequently in systemic sclerosis. We report a case of TC of the neck in a patient with systemic sclerosis. This case illustrates indications for the conservative management of spinal TC in patients with systemic sclerosis.

## Case presentation

A 67-year-old man presented for the evaluation of a hard neck mass associated with mechanical pain. He had a four-year history of systemic sclerosis, which has been treated with hydroxychloroquine, calcium channel blockers, and anti-CD-20 (rituximab) infusions. Additional medical history includes controlled hypertension, mixed hyperlipidemia, and restless leg syndrome.

The patient first noticed asymmetric Raynaud’s phenomenon and pigmentation over his hands, with subsequent mild joint swelling, skin dryness, and tightness of hands, fingers, forearms, neck, chest, and face. Physical examination at that time revealed sclerodactyly, pitting scars on several fingertips, nail-fold capillary abnormalities with giant capillary loops and hemorrhages, and matted telangiectasia on the palms, face, and upper chest. No synovitis or tendon friction rubs were noted. Laboratory data revealed the presence of antinuclear antibodies (titer 1:320) and Scl 70 specific antibodies. Pulmonary function tests and transthoracic echocardiographic findings were within normal limits, with no evidence of a restrictive process or the elevation of right ventricular systolic pressure.

**Figure 1:**
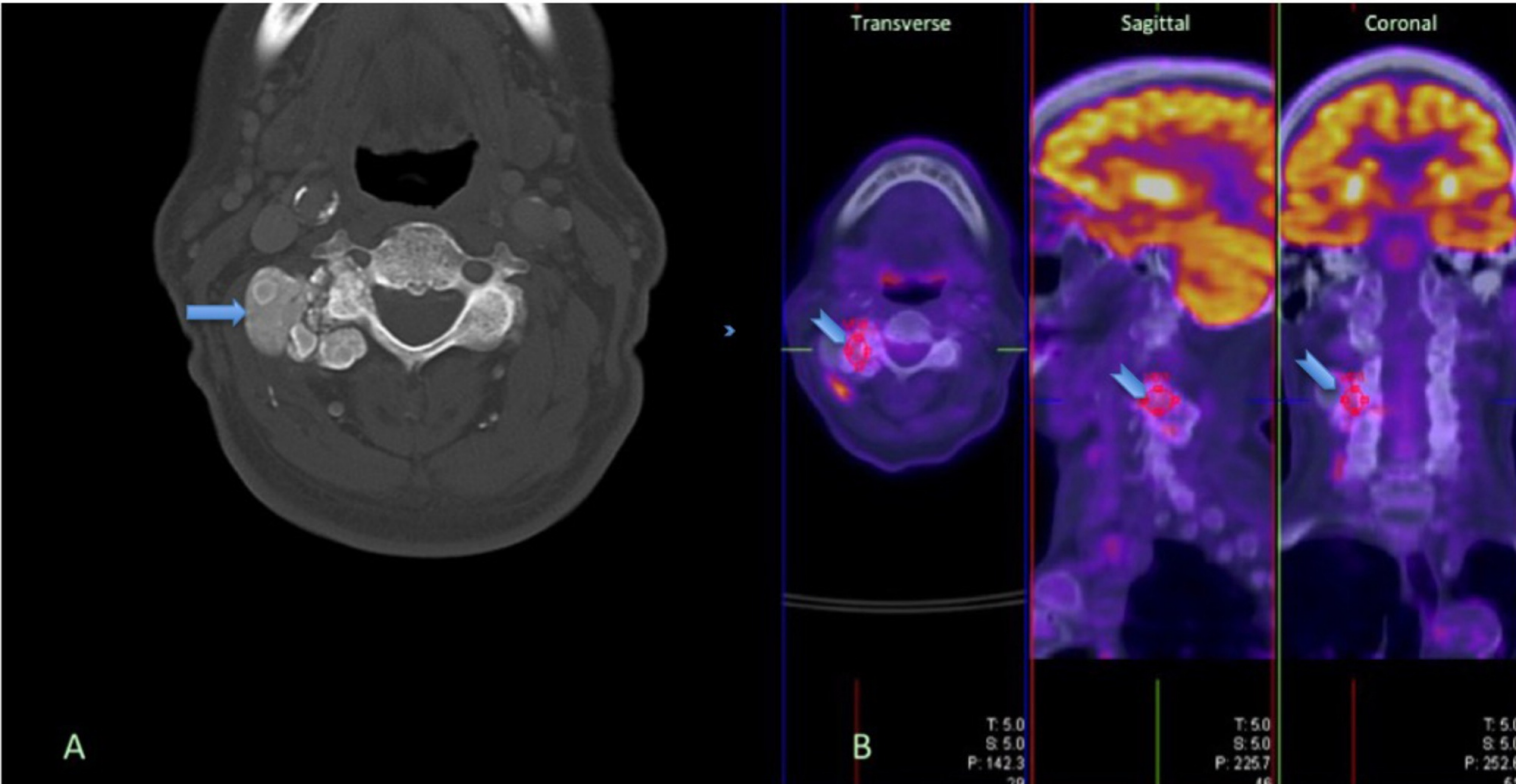
Cervical Tumoral Calcinosis A. Computer tomography (CT) of cervical calcinosis; B. Positron emission tomography-CT of mildly hypermetabolic calcified mass centered at the right C3-C4 facet joint

Two years later, he complained of a solitary mass on the right side of his neck, which was not present on previous imaging of the area and was slowly increasing in size. He denied neck trauma, dysphagia, odynophagia, or hoarseness but reported mechanical pain, which did not limit his daily activities. There were no other masses in the head, neck, or other parts of his body. Examination revealed a palpable neck mass in the right paramedian region that extended further laterally and anteriorly. Non-contrast computed tomography (CT) of the cervical spine from the skull base through the cervicothoracic junction, with multiplanar reformatted images and CT angiogram of the upper neck revealed a lobulated, calcified mass measuring approximately 3.5 x 2.1 cm centered at the right C3-C4 facet joint with an encroachment of the right transverse foramen. Guided biopsy (not shown) was negative for malignancy and amyloid and consistent with tumoral calcinosis. Positron emission tomography (PET)-CT found minimal hypermetabolic activity centrally within the mass and surrounding soft tissues most likely due to a recent biopsy. Figure 1 shows the CT (A) and PET-CT (B) aspects of imaging of the cervical spine TC in this patient.

Radical resection was not performed at the time, given his minimal symptoms and possibility of injury to the vertebral artery and the need for cervical fusion of his C3-C4 facet joint. Subsequently, his symptoms improved. Repeated examinations revealed no signs of spinal cord involvement. Repeated CT of the neck with and without contrast 18 months after the first CT showed no change in the size of the paraspinal mass. B-cell depleting treatment with rituximab was continued (1000 mg on Days 1 and 15 every six months). Sustained improvement of skin thickening and functional ability was noted. Interestingly, his previous, completely white hair turned gray, which involved not only the scalp but most of the body hair. Recent (March 2018) laboratory serum test values were as follows: creatinine 0.82 mg/dL, calcium 9.6 mg/dL, phosphorus 4.3 mg/dL, albumin 4.3 g/dL, alkaline phosphatase 84 U/L (normal range 38-126 U/L), parathormone (PTH) 37.6 pg/mL (normal range 7.6-77.2 pg/mL), 25-hydroxycholecalciferol 30.8 ng/mL (normal range 30-100 ng/mL). These values have been normal throughout the course of his disease.

## Discussion

TC is a rare syndrome. Fathi and Sakr [4] identified three categories of TC. Two of these categories represent primary entities while the third category is secondary to other systemic diseases. One primary category of TC (normophosphatemic familial TC) is characterized by normal calcium and phosphate metabolism and has recently been found to have a familial incidence and to be associated with a mutation of the gene encoding for the sterile alpha motif domain-containing protein 9 (SAMD9), which, in addition to TC, may play a role in the pathogenesis of inflammation and malignancy [4]. The second primary (hereditary) form of TC (hyperphosphatemic familial TC) is characterized by hypophosphaturia, hyperphosphatemia, and normal serum calcium levels, and is the consequence of loss-of-function mutations in one of three genes: fibroblast growth factor 23 (FGF23), which encodes for a phosphaturic protein; polypeptide N-acetylgalactosaminyltransferase 3 (GALNT3), which encodes for a transferase modifying FGF23; and Klotho, which encodes a coreceptor for FGF23 important in bone metabolism [4].

Secondary TC is encountered in a variety of diseases, including systemic sclerosis. Chronic kidney disease (CKD) is the most common condition associated with TC [4]. Hyperphosphatemia and secondary hyperparathyroidism are commonly associated with tissue calcinosis in CKD. An earlier report found hypophosphaturia, hypocalciuria, and elevated serum PTH levels in systemic sclerosis patients with calcinosis and proposed secondary hyperparathyroidism as the pathogenetic mechanism of this entity [5]. However, calcinosis in systemic sclerosis is currently considered to be a dystrophic calcification without overt abnormalities in the metabolism of calcium or phosphate [2-3]. The finding of normal serum levels of calcium, phosphorus, PTH, and vitamin D in our patient is in agreement with this current view.

TC has various adverse effects on the well-being of patients, depending on the size of the calcified mass and particularly on its location [4]. TC of soft tissues next to the spine has received particular attention. In patients with systemic sclerosis or its variants, symptomatic TC has been reported in the lumbar [6-8], thoracic [9], and cervical areas [6,10-20]. Among the 35 cases of paraspinal TC in systemic sclerosis patients discussed in a literature review by Sambataro and co-authors [20], 26 were in the cervical area. The involvement of facet joints with symptomatic nerve compression has been reported in several cases [6,9,15,17,19], including our patient.

The treatment of TC is tailored to individual cases and depends on the cause, site, and severity of symptoms. A conservative management approach through phosphate depletion, acetazolamide, vinpocetine, sodium thiosulfate, or intravenous pamidronate has been used in secondary TC with variable success [4]. Surgical excision of the mass is required in some cases but is often associated with high recurrence rate and complications.

Pain is the most frequently reported symptom of systemic sclerosis patients with paraspinal TC. Neurological symptoms or signs indicative of spinal cord compression were detected in 19 of the 35 cases reviewed by Sambataro and collaborators [20]. Like in our patient, pain without neurological deficits has been managed conservatively [19]. Indications for the removal of the tumorous mass and various surgical procedures to stabilize the spine include severe pain not relieved by conservative measures and, more importantly, the compression of the spinal cord with neurological deficits that can progress. Patients should be closely monitored for spinal neurological deficits. Surgical decompression of the spine has been reported in several cases [6-8,10-12,14-17-18,20]. Postoperatively, an improvement of the neurological deficits was noted in the majority of the reported cases. However, one patient died with sustained quadriplegia three months after the surgical procedure [14] and a second patient with prolonged tracheal intubation postoperatively died in the immediate postoperative period from cardiac arrest following vomiting and food aspiration [15].

## Conclusions

Paraspinal TC in patients with systemic sclerosis can cause intense pain and severe neurological manifestations. The condition can be treated conservatively in the absence of neurological deficits. However, the potential of severe neurological sequelae calls for the monitoring of the clinical picture and periodic imaging of the spinal TC. Surgical decompression, which has had satisfactory outcomes in the majority of cases, should be attempted in subjects with severe pain not responding to conservative measures or with neurological deficits attributed to spinal TC.
